# Calcium Dynamics in Dendrites of Hippocampal CA1 Interneurons in Awake Mice

**DOI:** 10.3389/fncel.2019.00098

**Published:** 2019-03-15

**Authors:** Ruggiero Francavilla, Vincent Villette, Olivier Martel, Lisa Topolnik

**Affiliations:** Department of Biochemistry, Microbiology and Bio-informatics, Faculty of Science and Engineering, Neuroscience Axis, CHU de Québec Research Center (CHUL), Laval University, Québec, PQ, Canada

**Keywords:** GABAergic inhibition, interneuron, dendrite, calcium, excitatory current, regenerative activity, behavior

## Abstract

Hippocampal inhibitory interneurons exhibit a large diversity of dendritic Ca^2+^ mechanisms that are involved in the induction of Hebbian and anti-Hebbian synaptic plasticity. High resolution imaging techniques allowed examining somatic Ca^2+^ signals and, accordingly, the recruitment of hippocampal interneurons in awake behaving animals. However, little is still known about dendritic Ca^2+^ activity in interneurons during different behavioral states. Here, we used two-photon Ca^2+^ imaging in mouse hippocampal CA1 interneurons to reveal Ca^2+^ signal patterns in interneuron dendrites during animal locomotion and immobility. Despite overall variability in dendritic Ca^2+^ transients (CaTs) across different cells and dendritic branches, we report consistent behavior state-dependent organization of Ca^2+^ signaling in interneurons. As such, spreading regenerative CaTs dominated in dendrites during locomotion, whereas both spreading and localized Ca^2+^ signals were seen during immobility. Thus, these data indicate that while animal locomotion is associated with widespread Ca^2+^ elevations in interneuron dendrites that may reflect regenerative activity, local CaTs that may be related to synaptic activity become apparent during animal quiet state.

## Introduction

Neuronal dendrites exhibit a large variety of voltage- and ligand-gated ion conductances and, therefore, may operate as independent signaling devices (Branco and Häusser, 2011). Calcium (Ca^2+^) signaling represents an important aspect of dendritic integration. It may have different spatial and temporal ranges of action, and can exert various functions from induction of synaptic plasticity and local tuning of neuronal firing to the regulation of the expression of genes involved in neurodegenerative processes (Verkhratsky, 2005; Higley and Sabatini, 2008; Camiré and Topolnik, 2012; Camiré et al., 2012; Topolnik, 2012). Due to methodological limitations, our current knowledge about the functional organization of dendritic Ca^2+^ signals stems mostly from experiments on glutamatergic principal cells (PCs; Regehr and Tank, 1990; Markram and Sakmann, 1994; Schiller et al., 1997; Golding et al., 2002; Losonczy and Magee, 2006; Sheffield and Dombeck, 2015; Sheffield et al., 2017). In hippocampal CA1 area, the dendrites of PCs express voltage-gated Ca^2+^ and sodium (Na^+^) channels and NMDA receptors. These mechanisms are involved in generation of dendritic regenerative activity in form of back-propagating somatic action potentials (bAPs) and local Ca^2+^ and Na^+^ spikes generated in single or multiple dendritic branches (Spruston et al., 1995; Magee and Johnston, 1997; Ariav et al., 2003; Gasparini et al., 2004; Losonczy and Magee, 2006; Grienberger et al., 2014). Activated by spatially and temporally coincident patterns of activity, these mechanisms may provide for membrane depolarization and supralinear Ca^2+^ signal required for induction of Hebbian forms of synaptic plasticity and important for place field firing (Magee and Johnston, 1997; Golding et al., 2002; Sheffield and Dombeck, 2015; Sheffield et al., 2017).

Local circuit GABAergic inhibitory interneurons in hippocampal regions control the integration and transfer of information during different network and behavioral states (Klausberger and Somogyi, 2008; Somogyi, 2010; Pelkey et al., 2017). These cells exhibit a large repertoire of voltage- and ligand-gated Ca^2+^ mechanisms, which are regulated differentially by changes in pre- and postsynaptic activity, and provide a means for a highly dynamic and versatile regulation of synaptic plasticity (Topolnik et al., 2005, 2006, 2009; Evstratova et al., 2011; Camiré and Topolnik, 2014; Hainmueller et al., 2014). In most interneurons, bAPs and, accordingly, the Ca^2+^ transients (CaTs) evoked by bAPs (bAP-CaTs) decline with distance from the soma due to a large K^+^ and low Na^+^ channel density (Aponte et al., 2008; Hu et al., 2010; Evstratova et al., 2011; Camiré and Topolnik, 2014). This means that only proximal synapses may be influenced by bAPs and are likely to exhibit the spike-timing-dependent forms of plasticity (Sjöström et al., 2008). This situation, however, may change rapidly, dependent on the level of on-going activity. In fact, bAP-CaTs can be boosted locally on a different time scale by the activation of additional voltage- and ligand-dependent Ca^2+^ mechanisms (Topolnik et al., 2009; Evstratova et al., 2011; Chiovini et al., 2014). Moreover, some CA1 interneurons, for example fast-spiking parvalbumin-expressing cells (including basket and bistratified cells) can exhibit large amplitude Ca^2+^ elevations in the absence of voltage-dependent dendritic mechanisms. In particular, we showed that Ca^2+^ entry through GluA2-lacking Ca^2+^-permeable AMPA receptors (CP-AMPARs) followed by Ca^2+^ release from internal stores is important for generating the supralinear Ca^2+^ signals, which control the direction of long-term plasticity at excitatory synapses located distally (Camiré and Topolnik, 2014). Other cell types, for example the CA1 *oriens lacunosum-moleculare* (OLM) cells, may have a relatively high density of Na^+^ channels in their dendrites and, subsequently, exhibit more wide-spread back-propagation of somatic APs (Topolnik et al., 2009) or even dendritic Na^+^ spike initiation (Martina et al., 2000), which can be tightly controlled *via* dendritic inhibition (Tyan et al., 2014; Francavilla et al., 2015). Taken together, these studies reveal a highly dynamic nature of dendritic Ca^2+^ signaling in interneurons. Yet, *in vivo* dendritic Ca^2+^ activity in these cells received little attention (Katona et al., 2011; Chiovini et al., 2014), and its regulation and functional significance during different patterns of network oscillations and behavioral states remain to be explored in details.

To begin examining the functional aspects of interneuron dendritic computations *in vivo*, here we performed two-photon Ca^2+^ imaging in CA1 *oriens/alveus* (O/A) interneurons of awake head-fixed animals running on a treadmill. We found that dendritic CaTs (dCaTs) exhibit the behavior-state fluctuations, such as regenerative activity during locomotion and dendrite-autonomous local signals during immobility. This state-dependent Ca^2+^ signaling suggests that distinct forms of synaptic plasticity can be induced in interneurons of awake mice during different behavioral states.

## Materials and Methods

### Mouse Surgery and Training

Experiments were performed on male C57BL/6 mice (P50–70) according to the procedures approved by the Animal Protection Committee of Université Laval (protocol #15-097-1). Mice were anesthetized deeply with ketamine/xylazine mixture (10/100 mg/kg) and fixed in a stereotaxic frame. A small (~0.5–1.0 mm) craniotomy was made over the hippocampus (AP: 2.1, ML: 1.8). For single cell dendritic imaging a low titer AAV1.Syn.GCaMP6f.WPRE.SV40 (Penn Vector Core) was diluted [1:4 in phosphate buffer saline (PBS, Gibco)] and injected (one injection of 100 nL) at a depth of ~1,250 μm below the dura surface, resulting in the expression of GCaMP6f in a sparse population of CA1 neurons (Chen et al., 2013).

After 4–6 days of recovery, a 3-day water restriction procedure was applied (0.8–1.0 mL/day) followed by a hippocampal window and head-plate implantation surgery (as described in Dombeck et al., 2010; Villette et al., 2017). Briefly, a bottom glass cannula (2 mm diameter) was inserted on top of dorsal hippocampus after cortex aspiration and secured with kwick-sil at tissue interface and Superbond at the skull level. Head plate was oriented using a 4-axis micromanipulator (MX10L, Siskiyou) and fixed with several layers of Superbond and dental cement (Villette et al., 2017). For dorsal hippocampus, a 7–13° medio-lateral angle was applied. Mice were allowed to recover for several days with post-operative pain killer treatment (Buprenorphine, 0.1 mg/kg, 48 h).

Behavioral handling with head fixation and training in a circular treadmill system (one ~10–15 min session per mouse per day) began ~5 days after window implantation and continued until mice routinely ran back and forth and demonstrated stable running speed values as described previously (Villette et al., 2017). Mouse locomotion speed and direction on the treadmill were monitored using an optical quadrature encoder (HEDS-5645#A06, Avago Technology). The immobility periods were determined as periods with no animal motion for at least 3 s. The locomotion periods were defined as periods of animal locomotion with a speed >2.0 cm/s for at least 3 s. Data was recorded using a Digidata1440A (Molecular Devices) data acquisition system (Clampex 10.2), which allowed synchronizing the animal speed and two-photon image frame timing (using the external trigger at 10,000 sampling frequency) and an AxoScope software (v10.5, Axon Instrument).

### Two-Photon Imaging of Interneuron Soma and Dendrites

Two-photon imaging was performed using Leica SP5 two-photon confocal microscope, the Ti:Sapphire laser (Chameleon Ultra II, Coherent) tuned to 900 nm and a 25× objective (0.95 NA, 2.5 mm working distance, Leica Microsystems). The laser power was modulated using a Pockels cell and reached at the sample (after the objective) 10–75 mW. Green GCaMP6f fluorescence was routed to external photomultiplier tubes (PMTs) (non-descanned detectors, Leica Microsystems). The Leica LAS software was used for microscope control and image acquisition. Image series (128 × 128 pixels, 0.5 ms per line field of view of 207 × 207 μm) in each plane were acquired at 47 Hz in single plane acquisitions. Imaging sessions lasted up to 30 ± 15 min and then the mouse was placed back in its home cage. Ca^2+^ imaging time-series were followed by z-series from each cell to obtain a detailed information on the imaged dendrites using the following parameters: 1 μm z-stack step size, 512 × 512 pixels per frame, 1 ms/line. The interneuron cell bodies were typically located 20–70 μm below the alveus surface.

### Data Analysis and Statistics

Analysis was performed using IgorPro (v.6.3, Wave Metrics) and custom scripts written in MatLab (The MathWorks). The time-series were motion corrected using whole frame cross-correlation, as described previously (Dombeck et al., 2010; Villette et al., 2017). Only dendrites that could be unambiguously traced back to soma were included in the analysis. Dendritic branches were traced off-line using Leica LAS software (*n* = 10 interneurons; 11 cells were excluded from the analysis because of a high brain motion during morphological z-series preventing accurate dendrite tracing). The dendritic distance to soma was calculated along the dendrite using the maximal projections of morphological z-series.

For CaT analysis, regions of interest (ROIs) were drawn manually on the mean soma or dendrite images following the outline of the structure of interest, and traces of ΔF/F vs. time were generated for each ROI as previously described (Villette et al., 2017; Francavilla et al., 2018). All CaTs occurring during immobility or consistent runs (speed >2 cm/s; longer than 3 s) were included in the analysis. Both average CaTs for the entire period of immobility or run as well as peak CaTs during individual events were analyzed. The local event detection was performed semi-automatically using the TaroTools toolbox in IgorPro. First, all events with peak amplitude exceeding the mean ± 2 SD level of the ΔF/F trace were selected from both somatic and dendritic traces recorded simultaneously. Then ΔF/F traces obtained from soma and dendrites were aligned and local dCaTs were defined as those occurring in the absence of concomitant somatic CaTs (sCaTs) within a 25-ms window. Cross-correlation analysis in IgorPro was used for correlation of dendritic and sCaTs with speed. The data are presented as mean ± SEM. The sample size was determined in preliminary experiments in compliance with ethical guidelines to reduce the number of animals used. Statistical significance between groups was determined using a Student *t*-test (in case of normal data distribution) or Mann-Whitney test (if the distribution of data was not normal as reported by the Shapiro-Wilk test).

## Results

**Figure 1:**
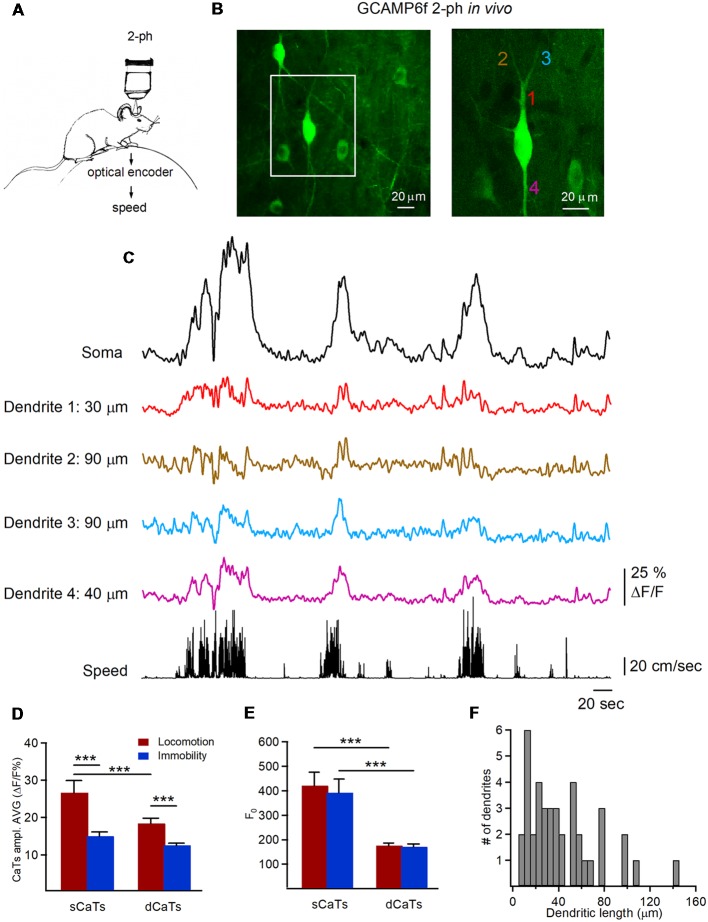
Two-photon imaging of dendritic Ca^2+^ transients (dCaTs) in hippocampal CA1 oriens/alveus (O/A) interneurons of awake mice. **(A)** Schematic of two-photon Ca^2+^-imaging and animal speed recording in awake head-restrained mice. **(B)** Two-photon images of the GCaMP6f-expressing interneurons in CA1 O/A (maximal projection of a 100-μm Z-stack) acquired at a high laser power to illustrate the cell morphology. The area depicted with a white square is expanded on the right. The numbers of different color indicate different dendritic branches (20 μm each), from which Ca^2+^ imaging data were obtained **(C)**. **(C)** Representative examples of soma and dCaTs, which were recorded in different dendritic branches and at different distance from the soma along with the animal speed (bottom black trace). Recordings of dCaTs were obtained from dendrites illustrated in (**B**; right). **(D)** Summary bar graphs of average amplitude for somatic CaTs (sCaTs) and dCaTs during locomotion and immobility, ***P < 0.001. **(E)** Summary bar graphs of basal fluorescence (F_0_) in soma and dendrites during locomotion and immobility, ***P < 0.001. **(F)** Summary cumulative histogram of the dendritic length (distance from the soma) at which recordings of dCaTs were obtained.

To study dendritic Ca^2+^ signals in hippocampal interneurons during different behavioral states, we performed chronic two-photon Ca^2+^ imaging of CA1 O/A interneurons labeled with a genetically-encoded calcium indicator GCaMP6f in head-restrained mice running on a circular treadmill (Figure 1A). Interneurons were identified based on the soma location within CA1 O/A and horizontally oriented dendrites located within the same focal plane (Figure 1B). Therefore, all imaging and analysis were performed only on horizontally oriented O/A interneurons and their dendritic arbors (128 dendritic segments of 10 μm each from 39 branches, *n* = 9 cells, 3.4 ± 0.3 min per interneuron per imaging session, one or two imaging sessions/cell, five mice) during immobility and locomotion. The imaging fields of view (207 × 207 μm) were selected to have in average 3–5 cells with clearly identifiable dendritic branches connected to the interneuron soma in the same focal plane (Figure 1B). The imaged dendritic branches had a mean length of 43.3 ± 5.1 μm from the soma (range: 7–145 μm; typical dendrite length in O/A interneurons ~200 μm) with a mean of 3.0 ± 0.6 branching points (range: 0–8).

According to previous reports, sCaTs result mainly from the AP firing, where the number of underlying APs correlates with fluorescence change (Kerr et al., 2005; Greenberg et al., 2008; Tian et al., 2009; Chen et al., 2013; Sheffield and Dombeck, 2015; Sheffield et al., 2017). Thus, in line with previous reports on the phase-dependent recruitment of different types of O/A interneurons during locomotion and immobility (Lapray et al., 2012), most O/A interneurons showed sCaTs throughout different behavior states (Figure 1C). Moreover, the mean sCaTs were significantly higher during locomotion than during immobility (*P* < 0.001; Mann-Whitney test; Figure 1D), consistent with phase-coupled rhythmic recruitment of O/A interneurons during theta oscillations associated with locomotion (Lapray et al., 2012; Katona et al., 2014; Lovett-Barron et al., 2014). This difference in the amplitude of sCaTs between the two behavioral states was not due to different basal somatic fluorescence (F_0_; Figure 1E).

Similar to sCaTs, dCaTs were detected during both locomotion and immobility (Figure 1C). Most of our recordings have focused on proximal dendritic branches (5–50 μm from the soma, 59%) made by primary dendrites, with the rest being secondary and tertiary dendritic branches extending up to 145 μm from soma (Figure 1F). While the basal fluorescence F_0_ was similar in dendrites during locomotion and immobility (Figure 1E), the amplitude of individual dCaTs varied across cell, individual dendritic segments and behavioral states (Figure 1C). The latter could not be associated with inter-cellular difference in GCaMP6f expression as variance in the basal fluorescence (F_0_) was similar in soma and dendrites when compared between different cells and behavioral states (sCaT_Loc_-F_0_: CV = 0.41, sCaT_Imm_-F_0_: CV = 0.45, dCaT_Loc_-F_0_: CV = 0.39, dCaT_Loc_-F_0_: CV = 0.39; *n* = 9 cells). Overall, like in soma, the average amplitude of dCaTs was significantly higher during locomotion (dCaT_Loc_: *n* = 128 segments/10 cells; dCaT_Imm_: *n* = 112 segments/nine cells; *P* < 0.001; Mann-Whitney test; Figure 1D), but it was also considerably smaller than in soma (*P* < 0.001; Mann-Whitney test; Figure 1D).

**Figure 2:**
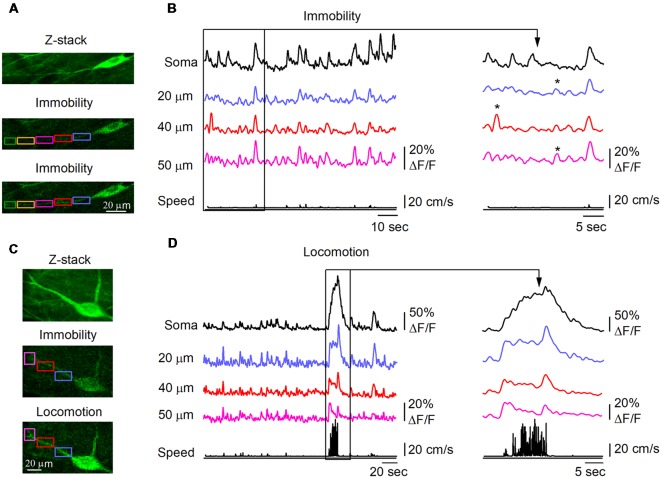
Behavior state-dependent CaTs in dendrites of O/A interneurons. **(A)** Representative two-photon images showing the interneuron morphology (top, maximal projection of a 10-μm *Z*-stack) and Ca^2+^ signals in soma and dendrites during immobility. Rectangle areas of different color indicate the dendritic regions of interest (ROIs) where dCaTs were analyzed. **(B)** Example traces of sCaTs and dCaTs recorded in some ROIs depicted in **(A)** during immobility along with the animal speed (black bottom trace). Local dCaTs that were not detected in soma are shown expanded on the right (labeled with stars). Panels **(C,D)** are the same as **(A,B)** but recordings were obtained for a second neighboring cell during locomotion period. CaTs recorded during locomotion are also expanded on the right.

**Figure 3:**
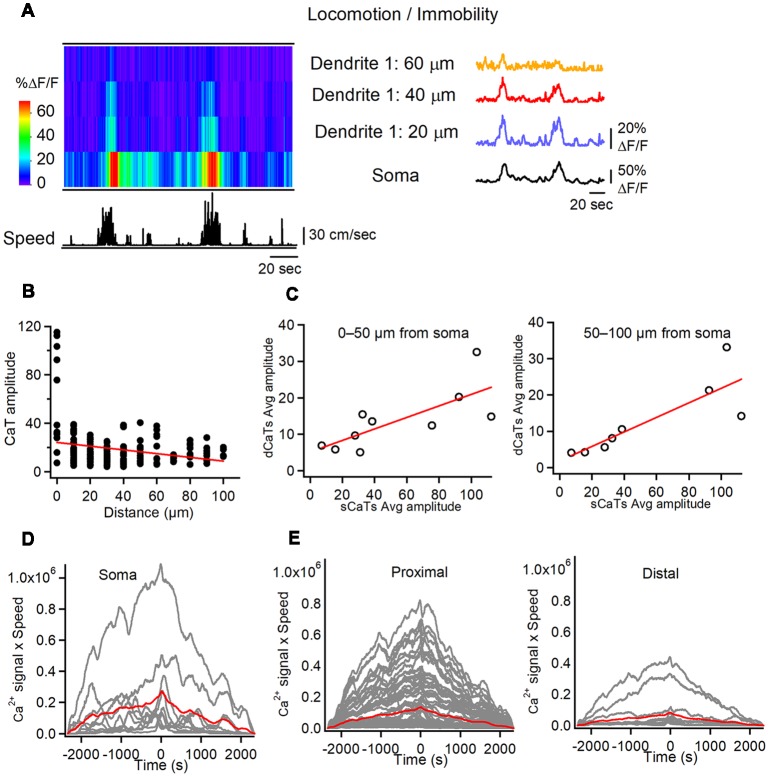
Soma-dendrite coupling in O/A interneurons during locomotion. **(A)** Representative heat-map of soma-dendrite Ca^2+^ activity in soma and dendrites along with the animal speed trace (left) and corresponding CaTs recorded in soma and dendrites during locomotion/immobility periods. Note large amplitude sCaTs that propagate to proximal dendrites (<60 μm from soma). **(B)** Summary plot illustrating the average dCaT amplitude as a distance from soma in comparison to that of sCaT (distance = 0) in different cells during locomotion. Red line is a linear regression fit to the data, indicating a significant decline in CaT amplitude with distance from the soma. **(C)** Summary plots illustrating relationships between dCaTs and sCaTs in proximal (0–50 μm from soma; left) and more distal (50–100 μm from soma; right) dendrites. Red lines correspond to a linear regression fit to the data, indicating a significant positive relationship between the sCaT and dCaT amplitude during locomotion. **(D,E)** Cross-correlation functions between the sCaTs **(D)** and dCaTs (**E**, proximal vs. distal) with animal running speed. Red traces indicate the mean cross-correlation functions (*n* = 9 cells).

As CaTs in dendrites of O/A interneurons can result from activation of local excitatory inputs (Topolnik et al., 2005, 2006) and regenerative activity (spread of bAPs or local dendritic spikes; Martina et al., 2000; Topolnik et al., 2009), which can be characterized by different spatial extent (Sheffield and Dombeck, 2015), we examined the properties of CaTs during different behavioral states (Figures 2, 3). Consistent with activation of local postsynaptic glutamate receptors, we observed small amplitude dCaTs restricted to single dendritic microdomains, with no apparent spread to neighboring dendritic segments or soma, which were defined as synaptic dCaTs (peak amplitude: 12.3 ± 0.7% ΔF/F; spatial extent: 21.4 ± 10.4 μm, *n* = 10 cells; Figures 2A,B). However, more frequently we observed large amplitude dCaTs that occurred synchronously with sCaTs (peak amplitude up to 50.0% ΔF/F, *n* = 10 cells; Figures 2C,D) and could be detected in the entire recorded branch (up to 100 μm from soma; Figure 2D). These events were considered as regenerative that could be evoked either by bAPs or dendritic spikes or both.

We then compared dendritic Ca^2+^ activity during locomotion with that during immobility. During locomotion, both soma and dendrites showed Ca^2+^ elevations, which were simultaneously detected over large dendritic distances (up to 100 μm from soma; Figures 3A,B). Using sCaTs as a surrogate measure of AP firing (Dombeck et al., 2010; Chen et al., 2013), we explored whether somatic AP firing was associated with dendritic regenerative activity in interneurons. First, during many run episodes with somatic firing, spreading dCaTs were observed (Figure 3A). These events were similar in amplitude to local synaptic dCaTs (15.4 ± 0.7% ΔF/F) but showed a strong linear correlation with sCaTs (Figure 3C), and declined with distance from the soma (*r* = –0.25, Pearson correlation, *P* < 0.01, Figure 3B), indicating that they were likely evoked by bAPs. Furthermore, in six out of nine cells, somatic Ca^2+^ activity correlated well with animal running speed (Figure 3D). Similarly, all proximal (<50 μm from soma) and some more distal (50–100 μm from soma) dendritic branches in these cells showed a positive correlation between dCaT amplitude and the running speed (Figure 3E). Taken together, these data indicate that during locomotion, dendritic Ca^2+^ signals in O/A interneurons correlate well with somatic activity and animal speed.

**Figure 4:**
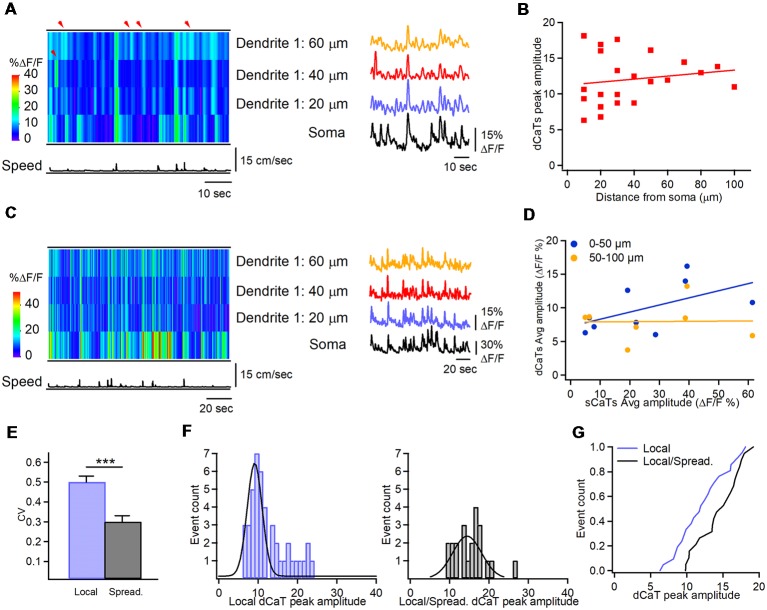
Soma-dendrite coupling during immobility. **(A)** Representative heat-map of Ca^2+^ activity in soma and dendrites along with the animal speed trace (left) and corresponding CaTs recorded in soma and dendrites during immobility, showing localized dCaTs that are not seen in soma (indicated with red arrowheads). **(B)** Summary plot showing the peak amplitude of dendritic local events as a function of distance from soma. Red line corresponding to a linear regression fit shows no significant relationship between the dCaT amplitude and distance from the soma. Panel **(C)** is the same as in **(A)** but during higher activity in the soma. Note the soma-dendrite spread of Ca^2+^ signals during some but not all events. **(D)** Summary plot illustrating relationships between dCaTs and sCaTs in proximal (0–50 μm from soma; blue) and more distal (50–100 μm from soma; yellow) dendrites. Lines are the linear regression fits to the corresponding sets of data, indicating no significant relationship between the sCaT and dCaT amplitude during immobility. **(E)** Summary bar graphs of the coefficient of variation for the peak amplitude of local vs. spreading events. Note a significantly higher variance of local events (***P < 0.001; *n* = 22 segments, nine cells). **(F)** Cumulative histograms of the peak amplitude for local dCaTs during immobility (left) and spreading dCaTs within the same dendritic segments during locomotion (right), with Gaussian fits to the data sets. **(G)** Cumulative distributions of dCaT peak amplitude for local and spreading events, recorded within the same dendritic segments during immobility and locomotion, respectively. Note a significantly highly amplitude of spreading events during locomotion.

During immobility, small amplitude dCaTs often occurred independently of soma and remained localized within individual dendritic segments, indicative of local synaptic activity (peak amplitude dCaTs: 12.0 ± 0.7% ΔF/F, *n* = 22 segments/10 cells; Figure 4A, shown with red arrowheads). These data suggest that local isolated dendritic activity, such as postsynaptic Ca^2+^ elevations, can be more frequently seen in interneurons during immobility than during locomotion state. Local CaTs were detected in both proximal and distal dendrites and had the same amplitude across dendritic tree (Figure 4B). Similar to dendritic Ca^2+^ activity during locomotion, some small amplitude dCaTs were recorded simultaneously with sCaTs likely in relation to somatic firing during immobility, and could spread between neighboring segments (Figure 4C). Also, some sCaTs did not invade dendrites, indicative of local inhibition (Figure 4C). Overall, during immobility, dCaTs showed no significant correlation with sCaTs (Pearson correlation coefficient: *r* = 0.3033; *P* = 0.2536; *n* = 18 segments/nine cells; Figure 4D), indicating that they were likely associated with activation of local Ca^2+^ mechanisms. The most distant dendritic sites were also the least coupled to soma (Figure 4D), consistent with previous observations *in vitro* (Camiré and Topolnik, 2014). Thus, in average, the soma-dendrite coupling likely *via* bAPs was stronger during locomotion, while the local synaptic activity dominated in interneuron dendrites during immobility.

Furthermore, during immobility state, dCaTs were more variable within a given cell. Overall, the variance in dCaTs was significantly higher in segments showing local events than in segments showing spreading events (Figure 4E). As some dendritic segments showed both local events during immobility and spreading dCaTs during locomotion, we assumed that summation of postsynaptic and bAP-CaTs could occur in such segments during locomotion. If this hypothesis is true, then larger dCaTs in such segments could be associated with coincident pre- and postsynaptic activity and induction of Hebbian forms of plasticity. To explore this idea further, we compared the amplitude of local dCaTs in such segments during immobility with that of spreading events during locomotion (Figures 4F,G). Our data showed that the mean amplitude of dCaTs in such segments increased significantly with locomotion (from 12.0 ± 0.7% ΔF/F to 15.2 ± 0.9% ΔF/F; *P* < 0.05; *n* = 22 segments; Mann-Whitney test; Figures 4F,G), with the majority of individual segments showing switch from small amplitude local events to considerable dCaTs (increase to 173.1 ± 17.3% of local dCaT, *n* = 13/22 segments), indicative of significant summation of dCaTs during spreading events. The remaining segments showed no change (98.5 ± 4.6% of local dCaT, *n* = 5/22 segments) or a slight decrease in dCaTs (decrease to 79.4 ± 8.3% of local dCaT, *n* = 4/22 segments) when compared to the local events. Taken together, these data point to a dominant summation of dCaTs during spreading events, but also highlight the segment-specific variability in local dendritic signaling, likely due to specific spatio-temporal arrangements of excitatory and inhibitory inputs converging onto interneuron dendrites.

## Discussion

Using high-resolution two-photon imaging, we explored dendritic Ca^2+^ activity in hippocampal O/A interneurons of awake mice during locomotion and immobility. Our data showed that, despite a large variability in dCaTs across the cells as well as within different dendritic segments of the same interneuron, dendritic Ca^2+^ activity in interneurons reflects the animal behavior, as different types of Ca^2+^ signals and dendrite-soma interactions were observed in specific behavioral states. As a rule, Ca^2+^ signals had larger amplitude and could invade the entire dendritic tree in the focus of observation during locomotion. While this type of Ca^2+^ activity was also present during immobility, the signal amplitude was significantly lower in both soma and dendrites. Moreover, a significant fraction of dendrites showed spatially restricted CaTs, which were not seen by soma. Based on these data, we propose a scenario in which high soma-dendrite coupling, likely due to bAPs or regenerative activity and associated dCaTs, may facilitate the spike-timing-dependent or Hebbian forms of synaptic plasticity in O/A interneurons during animal locomotion. In contrast, during animal quiet state, this type of activity can be reduced or even replaced by local dendritic Ca^2+^ signaling, which, if occurs at the hyperpolarized level of membrane potential, may facilitate the anti-Hebbian plasticity mechanisms.

Dendritic Ca^2+^ signals in interneurons are more complex than in PCs as they arise from activation of different mechanisms, including the postsynaptic Ca^2+^-permeable receptors, such as NMDA, CP-AMPAR (Goldberg et al., 2003a,b; Topolnik et al., 2005), kainate (Cossart et al., 1998, 2002) or α7 nicotinic acetylcholine receptors (Griguoli et al., 2013), different types of voltage-gated Ca^2+^-channels (VGCCs; Goldberg et al., 2004; Topolnik et al., 2009) and peri- and extrasynaptic group I metabotropic glutamate receptors (mGluR1/mGluR5; Topolnik et al., 2005, 2006; Camiré et al., 2012; Hainmueller et al., 2014). Activation of these Ca^2+^ sources usually triggers events of relatively small amplitude with different kinetic properties (from relatively fast to slow for ionotropic vs. metabotropic receptors), which are restricted to individual dendritic microdomains or branches. Consistent with activation of local excitatory inputs within single dendritic segments, spatially restricted postsynaptic dCaTs were observed in our study *in vivo* during both locomotion and immobility states. In addition, bAP-CaTs can be reliably evoked in proximal dendrites of CA1 interneurons *in vitro* *via* VGCCs and Ca^2+^ release (Topolnik et al., 2009; Evstratova et al., 2011). Our data *in vivo* indicate that such events may dominate during locomotion and, to a less extend, during immobility. Also, the Ca^2+^ -induced Ca^2+^ release (CICR) events of large amplitude can be generated in some interneurons at rest (e.g., fast-spiking cells; Camiré and Topolnik, 2014), and can be seen during immobility given an overall lower spiking activity of O/A interneurons (Lapray et al., 2012; Varga et al., 2012). In summary, many Ca^2+^ sources can interact in interneuron dendrites depending on the level of on-going network activity and the functional state of a given Ca^2+^ source, indicating that dendritic Ca^2+^ activity in interneurons *in vivo* may be even more diverse than *in vitro*.

Indeed, we demonstrate significant variability in interneuron dCaTs *in vivo*. First, we found that different dendritic segments of the same cell could demonstrate different types of dCaTs likely due to a high diversity of post- and extra-synaptic Ca^2+^ mechanisms expressed across dendritic arbors (Camiré and Topolnik, 2012; Camiré et al., 2012; Topolnik and Camiré, 2019) and a different degree of bAP propagation or bAP-CaT signal amplitude due to activity-dependent regulation of these processes *via* synaptic inhibition (Tyan et al., 2014; Francavilla et al., 2018) or mGluR5-dependent modulation (Topolnik et al., 2009). In addition, the dCaT variability could arise from different types of O/A interneurons sampled in our study, including somatostatin-expressing OLM, bistratified and long-range projecting cells (Sik et al., 1995; Halasy et al., 1996; Jinno et al., 2007) as well as horizontal basket (Maccaferri, 2005) or trilaminar (Ferraguti et al., 2005) cells. The detailed cell type-specific organization of dendritic Ca^2+^ signaling remains still to be examined in the *in vitro* and *in vivo* studies. However, regardless of the inter-cell and intra-cell variability, dCaTs in our study showed consistent behavior state-dependent organization, indicating that similar mechanisms may drive dendritic Ca^2+^ activity in different inhibitory cell types during a particular behavior state.

During locomotion, high power theta oscillations are detected in the CA1 hippocampus, with different types of O/A interneurons exhibiting rhythmic phase-dependent firing (Lapray et al., 2012; Varga et al., 2012; Katona et al., 2014). Dendrites of different types of O/A interneurons receive theta-modulated excitatory input from CA1 PCs, which almost linearly increase their firing rate with the animal speed (McNaughton et al., 1983; Czurkó et al., 1999; Buzsáki, 2002). Accordingly, analysis of somatic and proximal dendritic Ca^2+^ events revealed a good correlation between these signals and the animal speed in O/A interneurons. In addition, the local inhibitory inputs that terminate onto O/A interneuron dendrites are made by the type 3 interneuron-specific interneurons (IS3; Acsády et al., 1996; Chamberland et al., 2010; Tyan et al., 2014) and the long-range projecting vasoactive intestinal peptide (VIP)-expressing GABAergic neurons (VIP-LRPs; Francavilla et al., 2018). While IS3 cells may fire periodically during theta-run epochs, their activation is often delayed and is rather irregular (Luo et al., 2019), indicating that dendritic IS3 inhibitory input may not be efficient in controlling interneuron dendrites during locomotion. The VIP-LRPs are, in turn, the theta-off cells and do not participate in dendritic input modulation during theta (Francavilla et al., 2018). Thus, interneuron dendrites are likely disinhibited during theta-run epochs, which may facilitate the spread of bAPs or local spikes and generation of widespread dCaTs, as observed in our study.

During animal quiet state and consummatory behavior, large irregular activity (LIA) with periodic sharp-wave-associated ripples (SWRs, 120–250 Hz) are recorded in the CA1 hippocampus, with CA1 PCs firing mostly during SWRs. The IS3 and VIP-LRP cells are active during LIA but do not participate to SWRs (Francavilla et al., 2018; Luo et al., 2019), indicating that interneuron dendrites are mostly inhibited during animal quiet state with short periods of disinhibition and excitatory drive received during SWRs. Our findings of significantly reduced dCaTs during immobility are in line with these observations. It remains to be determined whether the spatially restricted dCaTs detected in our study are associated with SWRs.

Dendritic Ca^2+^ activity in interneurons has been consistently associated with induction of Hebbian and anti-Hebbian long-term potentiation (LTP; Perez et al., 2001; Lamsa et al., 2005, 2007; Topolnik et al., 2006; Topolnik, 2012; Camiré and Topolnik, 2014; Hainmueller et al., 2014). In this context, our observations of highly variable dCaTs in interneurons of awake mice imply that distinct forms of synaptic plasticity may be induced across different cells and synaptic inputs terminating onto specific dendritic segments. Given a prevalent regenerative dCaTs during locomotion, we propose that Hebbian LTP can be induced in O/A interneurons during locomotion, thus enhancing their somatic firing (Perez et al., 2001; Lamsa et al., 2005; Croce et al., 2010). During immobility, when the dendritic inhibitory drive to O/A interneurons is increased (Francavilla et al., 2018; Luo et al., 2019), the local membrane potential is likely hyperpolarized. Accordingly, the strong excitatory inputs arriving during SWRs and associated with local dCaTs may induce bidirectional plasticity depending on the Ca^2+^ sources involved (Lamsa et al., 2007; Griguoli et al., 2013; Camiré and Topolnik, 2014). This simplified scenario does not consider other potential mechanisms that may operate during different behavioral states, including the septo-hippocampal cholinergic, glutamatergic and GABAergic projections as well as modulation of local dendritic conductances. Additional studies will be required to directly reveal how different types of dendritic Ca^2+^ activity induce synaptic plasticity *in vivo* and which learning rules may be specific for interneurons in behaving animals performing cognitive tasks.

## Author Contributions

RF and VV performed the experiments. RF, OM and LT analyzed the data. RF and LT wrote the manuscript.

## Conflict of Interest Statement

The authors declare that the research was conducted in the absence of any commercial or financial relationships that could be construed as a potential conflict of interest.
